# Death of Dr. Cummin

**Published:** 1837-07-01

**Authors:** 


### 4. DEATH OF DR. CUMMIN.

This took place at his house in Great Russell Street, on the 10th of April, in the 37th year of his age.

Dr. Cummin was an able writer, a clever journalist, and, by the concurrent testimony of those who knew him best, an honest man. His lectures on forensic medicine were highly valuable, and much esteemed.

